# Raman spectroscopic peculiarities of Icelandic poorly crystalline minerals and their implications for Mars exploration

**DOI:** 10.1038/s41598-022-09684-x

**Published:** 2022-04-04

**Authors:** Victoria Muñoz-Iglesias, Laura Sánchez-García, Daniel Carrizo, Antonio Molina, Maite Fernández-Sampedro, Olga Prieto-Ballesteros

**Affiliations:** Centro de Astrobiología (CSIC-INTA), Madrid, Spain

**Keywords:** Biogeochemistry, Planetary science

## Abstract

In this work, we have analyzed natural samples collected at three hydrothermal areas of Iceland by Raman spectroscopy. The studied high-latitude regions are considered environmentally and mineralogically appropriate Martian analogues since they are rich in weathered basalts that have been altered by hydrothermalism to mineral phases such as silica, clay minerals, sulfates, oxides, and sulfur. The main objective of this work was to assess the relation of the spectroscopic signatures of alteration to hydrothermal processes and biomediation, considering previous studies focused on the detection of lipid biomarkers in the same samples. The recorded Raman spectra, taken with optical parameters similar to the ExoMars 2022 Raman spectrometer, showed structural modifications in all secondary minerals in the form of peak shifts (in the case of sulfur and clay minerals), changes in the relative ratio intensity (in anatase) and/or shape broadening (in sulfates and hematite). These results reveal the suitability of Raman spectroscopy to examine areas rich in water-altered minerals, where a mixture of crystalline and amorphous phases can co-exist. The detection of silica is singularly interesting since, on the one hand, it can imply the past existence of hydrothermal hot springs rich in nutrient and redox gradients and, on the other hand, provides excellent matrix for biosignature preservation. The data can be helpful as an astrobiological database for the forthcoming missions to Mars, where potential upwelling groundwater systems could have altered the mineral phases in a similar way to that observed in this work.

## Introduction

Current Mars surface mineralogy resulted from the planet’s endogenous and subsequent hydrologic and atmospheric activity varying along time. The planet history can be divided into three main periods relating to changes in environmental conditions. Although there is no unanimous consensus, several findings suggest that early Mars had the environmental conditions required to have supported the presence of water under and on the planet's surface^1,2^. For example, Chevrier & Mathè^3^ proposed a theory about the possible evolution of Mars. The oldest period, the Noachian era ranging from 4.1 to about 3.7 Gya ago^4^, was characterized by a thick H_2_O/CO_2_-rich atmosphere. The formation of significant amounts of clay minerals could have taken place through weathering processes of basaltic composition rocks^5^. In the next period, the Hesperian (3.7 to approximately 3.4 Gya), the favorable conditions for the presence of water would have decimated but sporadically recurred^6^. In contrast, the gradual loss of that atmosphere and an intense volcanic activity would have acidified the environment and led to the precipitation of part of the sulfates observed nowadays. Finally, in the most recent epoch, the Amazonian, the formation of additional poorly crystalline phases (mainly iron (oxy) hydroxides) could have taken place under strongly UV-radiation and oxidizing environment (with a predominance of H_2_O_2_, O_2_) at extreme dry and cold conditions. Therefore, these mineral types (clay minerals, sulfur, sulfates, and oxides) must be target matrices in the coming missions to Mars since they can give valuable information about the planet’s history.

The minerals formed throughout the Martian planetary history suffered later alteration processes caused by several environmental factors such as temperature, radiation and pressure^7,8^. In this last case, samples could be formed in relative high-pressure environments such as the potential past ocean floor, or have suffered a later pressurization through diagenesis after their formation (if they were buried at depths down to 5 km by lava flows, for instance), or be altered at very high-fast pressurizations by shocking impact events). Fornaro et al*.*^9^ performed a meticulous review about all these possible mineral alteration processes that can affect the preservation of organics. In that review, the authors explained the complexity of the interactions between minerals and organic molecules. The formers were able to act either as catalysts or, conversely, protectors of the organics against degradation. Also, water can accelerate the degradation of the potential biomarkers or act as a UV shield when in a solid state, absorbing the radiation. Hydrothermal silica-rich deposits on Mars were found by the rover Spirit. These deposits of opaline silica associated with volcanic materials were found in the form of both outcrops and regolith. Since hydrothermal systems are commonly related to microbial communities on Earth, this finding opened a new window for the possibility of the existence of a past Martian life^10^. Subsequent investigations of these Martian regions compared to similar hydrothermal systems on Earth, such as the hot springs at El Tatio in Chile, showed that the deposits found by Spirit have characteristic nodular and digitate structures similar to those found in El Tatio formed after a combination of both abiotic and biotic processes^11^.

Further evolution of areas affected by hydrothermalism can occur, including the inactivation of these processes and their burial by deposition of volcanoclastic or other sedimentary material. At the subsurface, the system becomes a radiation-protected and potentially more hydrated environment. Recent studies found evidence suggesting the possible existence of upwelling groundwater systems at both during Hesperian^12^ and nowadays, from the spatial correlation between recurring slope lineae source regions and multi-scale fractures^13^. In the first case, the authors observed sapping valleys (carved by groundwater activity) in north plane deeper basins, which intercepted the water-saturated zone during the Hesperian and exhibited signs of groundwater fluctuations. When looking for the surface mineralogy found at those zones, Pan et al*.*^14^ detected pervasive olivine, pyroxene and hydrated minerals (Fe/Mg smectite, chlorite, prehnite, and hydrated silica) despite local mineral heterogeneities. The hydrated minerals were concentrated in large craters, and the authors argued that the minerals could have been exhumed from deep basement rocks.

There is a need to make an extensive spectroscopic database using the parametric restrictions of Raman spectrometers that will be part of the instrumentation of future missions, such as the limitations in their optical systems. While the detection of clay minerals with a non-confocal Raman spectrometer is challenging due to the small grain size^15^, sulfates and oxides, among others, usually give intense Raman signals that allow a good mineral characterization^16^. Interestingly, there is extensive work relating the Raman signatures to biomediation on minerals, which we summarize and compare with our own spectra of natural mineral phases taken in two field campaigns to Iceland during 2017 and 2018.

Here, we wanted to advance spectroscopic technical strategies for exploring Mars and its mineralogy, with an eye on the search for hypothetical biomediated structures. Therefore, we aimed at studying the specific Raman spectra of analogous minerals that could have suffered alteration processes similar to those on Mars, since the most recent missions to the planet, ExoMars and Mars2020^17,18^, carry Raman instruments. Also, ExoMars rover drills (down to 2 m) will allow assessing the water content, textural information, and rough grain size of the minerals located at the Martian subsurface, with the joint aims of searching for any evidence of present or past biosignatures. It is expected that the data obtained in these missions contribute to select the best target sites for the next missions to gain deeper insights into the potential biogeochemical history and natural resources of the planet^19–21^. The better the databases elaborated previously on Earth, the more founded will be their signal interpretation during the mission operations. Raman spectroscopy is a powerful technique that can give relevant information about the sample, not just about its chemical composition but also its physical and crystalline states. However, the correct acquisition of these data is not an easy task, and previous studies at the laboratory with the assistance of additional characterization techniques are necessary. The technique has been evaluated in the laboratory for its more suitable use for astrobiological purposes^22^. Several works carried out a good performance in this regard by the development of techniques to enhance the Raman intensity signal of organic compounds by using surface-enhanced Raman spectroscopy (SERS)^23^ and studying with accuracy the Raman signature of biologically essential amino acids^24^. However, works with non-confocal spectrometers for space use, such as the one presented here, are scarce but required since it is necessary to determine the limitations of the current field equipment (see the multi-instrument approach performed to obtain a terrestrial analog spectral library by the ExoMars team at https://www.ptal.eu/final-review-ptal-project).

Iceland has been suggested as an analog for Mars in volcanology, petrology, glacial, and surface alteration processes (e.g.,^25^). Its petrology is mainly comprised of extrusive basaltic lava flows (with high Fe content similar to the displayed by the Martian basaltic rocks), forming a porous and water-rich media that is affected by intense hydrothermal activity. Mud and steam springs are abundant on the surface, with a wide pH range. This diversity provides that all the minerals mentioned in the first paragraph are found in Iceland, where we selected three hydrothermal areas to be explored in situ and to analyze in the laboratory by Raman and IR spectroscopies, XRD, and for the molecular distribution and stable-carbon isotopic composition of lipid biomarkers^26^. These three areas were selected because of their abundance of Fe-rich basalts common in the region, the oxidation conditions of their hydrothermal fluids, and the presence of extensive sulfate and phyllosilicates-rich deposits from basalt weathering. The first site was “Krýsuvík–Seltún” (63.90 N, 22.05 W), located in the center of the Reykjanes peninsula, and defined by extensive post-glacial lava fields, ridges of pillow lavas, pillow breccia and hyaloclastites, all basaltic in composition. The second place was “Hengill–Hveragerði” (64.05 N, 21.21 W), a geothermal area located in the extinct Hveragerði volcanic system, which connects with the active Hengill volcano by fissures swarms. In this area, rocks are subglacially forming hyaloclastites and interglacial basaltic lava flows. “Námafjall” (65.64 N, 16.82 W) was the third explored site, a geothermal area located east of Lake Mývatn, at the base of a volcanic mountain. Subaereal hydrothermal activity is reflected in all these locations through mud pools, fumaroles, hot springs, and hot grounds.

For this research, 47 samples were analyzed by Raman spectroscopy. Also, IR spectroscopy and XRD measurements assisted in the sample characterization, since not all the mineral phases are detected with each technique. In the Methods section, details are included on the sampling sites (i.e., coordinates, field description, in situ temperature, and pH) and the characteristics of the equipment used in the laboratory for sample characterization.

## Results and discussion

### Icelandic samples characterization

The mineralogy of all the samples collected at the three studied areas of Iceland, analyzed at the laboratory, was determined by XRD, Raman, and IR spectroscopies (Supplementary Table S1 online). Hence, 14 samples were collected at “Krýsuvík–Seltún”, 8 from rocks (hereafter referred to as “R”), 2 from microbial mats (hereafter referred as “MAT”), 1 from hot spring precipitation (hereafter referred as “HP”), 2 from active fumaroles (hereafter referred as “AF”) and 1 from a mud pot (hereafter referred as “MP”). At “Hengill–Hveragerði”, 25 samples were selected, 15 from R and 10 from MAT. And, finally, at “Námafjall” we collected 8 samples, 4 from inactive fumaroles (hereafter referred to as “IF”), 2 from MP and 2 from HP. Each sample is named with the initial of the site where it was collected (“K” for “Krýsuvík–Seltún”, “H” for “Hengill–Hveragerði”, and “N” for “Námafjall”), then the substrate type (R, AF, IF, MP or MAT), and finally the number in sequential order (1, 2, 3…). Thus, for instance, the first sample collected in “Hengill–Hveragerði” from a rock was named H-R-1. The equivalence with the nomenclature of the samples previously analyzed in Sánchez-García et al*.*^26^, where we focused on the fingerprinting molecular and isotopic biosignatures, can be seen in Table S2 of the Supplementary Online Information.

### Particular Raman spectroscopic signatures

Attending exclusively to Raman spectra, we explain below the main mineral phases measured in the three hydrothermal regions in Iceland: sulfur, hematite, anatase, sulfates, and potentially clay minerals. We compare them with the literature and the free available RRUFF database (http://rruff.info/^27^). We show the best and more representative Raman spectra obtained from all the samples analyzed for each particular study case.

The highest vibrations of native sulfur, caused by the internal modes of the ring S_8_, are located around 225 and 475 cm^−1^^28^. Hematite is recognizable in the low frequencies’ modes around 225, 295 and 410 cm^−1^, and also at the Raman signature at 1320 cm^−1^ caused by a 2nd harmonic vibration^29–31^. Anatase is identified by its vibrational modes at 395, 510 and 635 cm^−1^^32^. Sulfates are well characterized and distinguishable by their sulfate (SO_4_^2–^) stretching vibration mode^33^. For instance, in halotrichite, it appears around 990 cm^−1^, in epsomite at 985 cm^−1^, in gypsum at 1010 cm^−1^, while in natroalunite at 1025 cm^−1^ (see Fig. S1 of the Supplementary Information).

As kaolinite or montmorillonite, clay minerals can be identified by their fundamental modes below 1000 cm^−1^^34^. However, although we knew they were abundant in the samples studied by XRD and IR spectroscopy, their weak Raman signals usually appeared hidden by other mineral phases and fluorescence.

Additionally, organic compounds belonging to photosynthetic organisms (e.g., pigments from phototrophs such as algae or cyanobacteria) were also detected in some samples by Raman spectroscopy (see Fig. S2 of the Supplementary Information). The Raman spectrum of chlorophyll was observed by three characteristic signals around 1000, 1150, and 1510 cm^−1^ resulting from C–CH_3_, C–C, and C=C stretching vibrations, respectively^35^.

**Figure 1:**
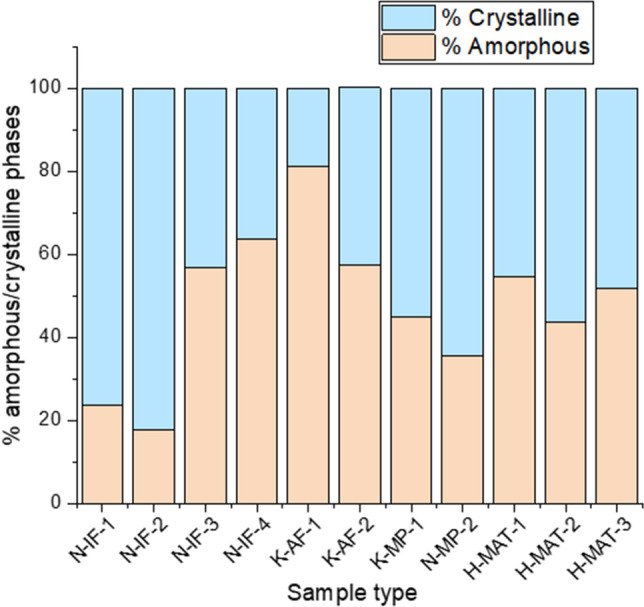
Index of crystallinity determined by XRD of selected samples found in a diversity of substrates, where previously the molecular distribution and stable-carbon isotopic composition of lipid biomarkers were determined^26^. IF: inactive fumarole, AF: active fumarole, MP: mud pot, MAT: microbial mat.

Spectroscopic signatures of the mentioned minerals and organic forms were detected in different environments of study, e.g.*,* both inactive and active fumaroles (IF/AF), mud pots (MP) and microbial mats (MAT). Anatase and native sulfur were measured in all the settings. Both minerals have intense Raman signatures in the wavelength of the laser used (i.e., 532 nm) that even can obstruct the detection of other mineral phases when located at their side in the same sample (see Fig. 5 of^26^). As mentioned, we focused our attention on five mineral types: (a) native sulfur, (b) hematite, (c) anatase, (d) sulfates, and (e) clay minerals, to study the relationship between structural disorder (represented in Raman shift and peak broadening) and biological activity and/or hydrothermal processes. To help with our interpretations, we used previous semi-quantitative XRD analyses of the samples that approached the percentage in each sample of amorphous and crystalline phases (Fig. 1). These hydrothermal systems are also rich in primary amorphous phases, such as hydrated silica. The lack of a clear relationship between the crystallinity and the type of substrate (IF, AF, MP, MAT) suggests that amorphization of samples can be caused by both inorganic and organic processes, that is, from hydrothermalism to biomediation, respectively. Subsequent physical and/or chemical weathering could contribute to the loss of crystallinity of the mineral phases. Physical weathering involves mineral alteration by temperature–pressure changes. Chemical weathering can be caused by various agents depending on the environment surrounding the mineral. In this sense, a mineral can undergo carbonation, oxidation, hydration, hydrolysis and/or acidification processes that can promote its transformation to a new mineral phase. On the other hand, organic processes are those that involve biological activity on mineral surfaces, altering the crystalline structure through the use of chemical elements of the mineral^36^.

#### Native sulfur

**Figure 2:**
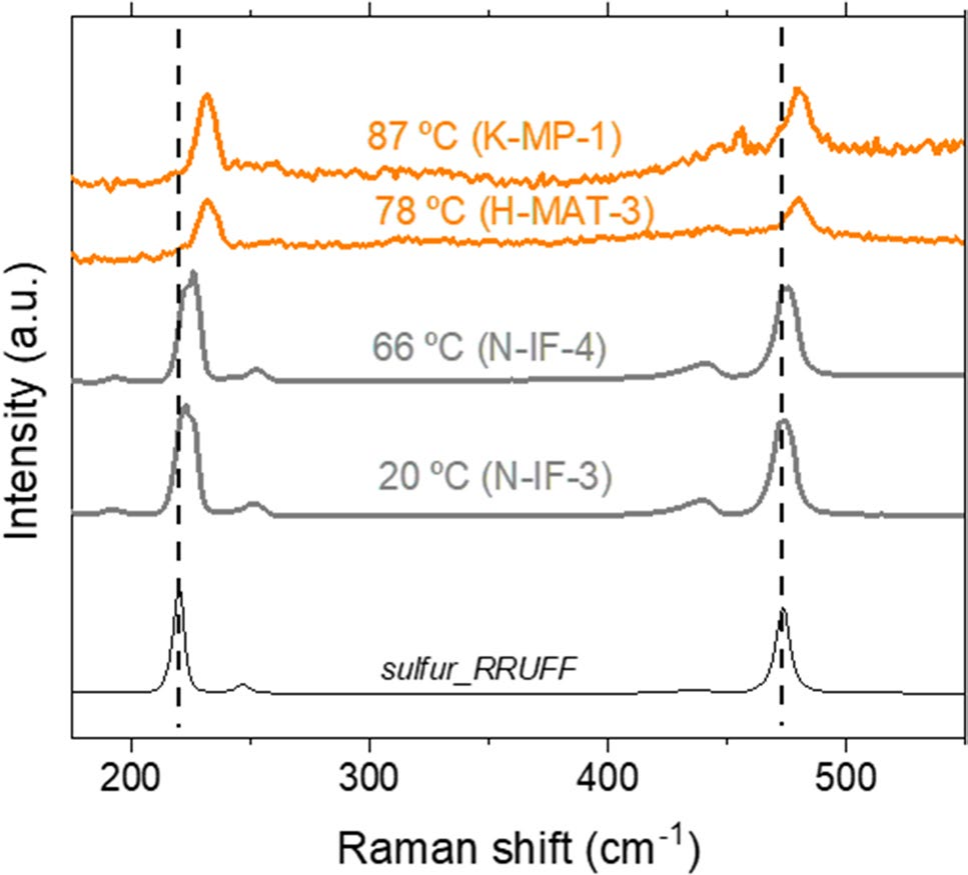
Raman spectra of selected sample spots where elemental sulfur was detected and its relationship with temperature. At the bottom, the sulfur Raman spectra from the RRUFF database are shown to compare and identify the shifts of the vibrational modes of sulfur according to the increase in temperature. For sample naming codes see Supplementary Table S1 online.

Native sulfur was widely detected in samples located in all the sites and substrates studied (see Supplementary Table S1 online). We observed a relationship between the band shifts of the native sulfur and the temperature of the samples. While in samples below 70$^\circ$C, such as N-IF-3 and N-IF-4, the vibrational modes fit well with those indicated at the RRUFF database, in the samples above 75$^\circ$C, such as H-MAT-3 and K-MP-1, as we mentioned before, the peaks suffered a Raman shift from 225 and 475 cm^−1^ to 231 and 479 cm^−1^, respectively (Fig. 2). This change can be an indication of structural alteration of the mineral. The spectra of these high-temperature samples (i.e., microbial mats and mud pots at a temperature above 75$^\circ$C) also showed a slight band broadening and intensity reduction that suggests a higher level of amorphization of the crystal structure.

Both groups differed *de visu* in color. While the low-temperature samples showed yellow color, those samples at high temperature were whiter. All these characteristics helped us to guess that we were observing different sulfur allotropes. As α-sulfur is commonly yellow, it is probably the allotrope present in the low-temperature samples. It transforms in the β-allotrope at temperatures above 95$^\circ$C, also yellow, but it is not as frequent in nature since it is not stable below that temperature and converts rapidly to α-sulfur. γ-sulfur, however, is a pale-yellow allotrope forming needles, which fits quite well with our samples at high temperatures. It is formed by S_8_ rings as the other allotropes but is packed differently and, interestingly, it forms by slow cooling of sulfur that was previously molten above 150$^\circ$C^37,38^. This situation can be easily achieved during a hydrothermal event. In agreement with previous works^39^, the Raman shifts observed in our Icelandic samples support the interpretation that the high-temperature sulfur is in the γ form.

Based on previous studies^40,41^, the displacement of the Raman peaks of the high-temperature sulfur can be associated with biological activity. Eder et al*.*^40^ measured intracellular sulfur in *Candidatus Magnetobacterium bavaricum* (*Nitrospirae*). They interpreted the sulfur-attributed Raman peaks in the uncultured bacteria as poor crystalline α-S_8_ and considered the amorphization as a potentially biomediated process/biologically induced process. The bacteria studied are found in worldwide aqueous environments^42^. Nims et al*.*^41^ characterized the intra- and extracellular content of sulfur in mats of sulfur-oxidizing γ-Proteobacteria (*Thiothrix*) by Raman spectroscopy. They observed by a fine analysis that the intracellular sulfur consisted of an amorphous cyclooctasulfur (S_8_), while the extracellular was formed by a mixture of the three allotropes α-, β-, and γ-S_8_.

With the technical parameters used in our study (i.e., related to a spectral resolution of 5 cm^−1^) and using a larger Raman laser spot size, obtaining a comparable level of detail was unaffordable. Still, we were able to distinguish between two sulfur forms that suggest a different origin for each allotrope, with the allotrope with high-frequencies Raman shifts (found in samples at field temperature above 75$^\circ$C, i.e., H-MAT-3 and K-MP-1) being potentially related to biological activity according to the high temperature-presence of biosignatures association made by^26^. We can suggest that related-hydrothermalism episodes could favor the formation of the γ allotrope instead of the α form.

#### Hematite

Although less abundant than sulfur, as mentioned above, hematite characteristics Raman modes around 225, 295 and 410 cm^−1^, and 1320 cm^−1^ were measured in samples from all the sites (see Supplementary Table S1 online). However, an additional broad band appeared in all the recorded spectra centered at around 800 cm^−1^ with a shoulder at 660 cm^−1^. We tentatively assign the band at 800 cm^−1^ to opal^26,43^.

Broad bands can be an indication of loss in crystallinity. However, the band could also be caused by other minerals or impurities at concentrations below the detection limits of the equipment used.

**Figure 3:**
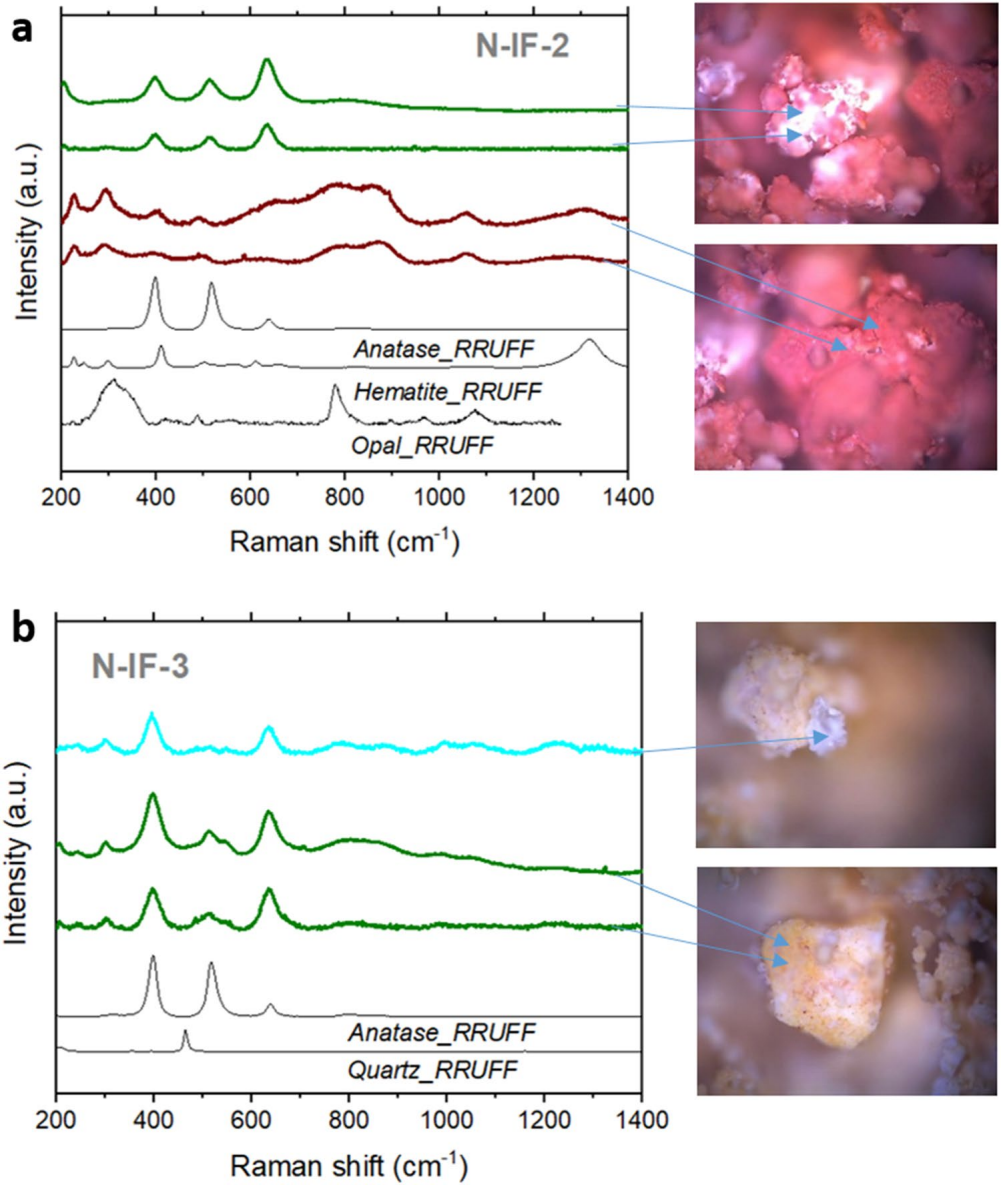
(**a**) Raman spectra at several spots of inactive fumarole N-IF-2, where anatase (green spectra) and hematite (red spectra) were detected. (**b**) Raman spectra at several spots of inactive fumarole N-IF-3, where mainly anatase was measured. The high Raman signal of anatase masked the quartz previously detected by XRD. Blue arrows indicate the spot where the spectra were obtained.

Kilias et al*.*^44^ found structural-morphological evidence of biomediation in hematite through Raman spectroscopy, relating their extracellular polymeric substances to past microbial mat activity and a white smoker-type seafloor hydrothermal environment. In their samples, microstromatolites composed of hematite and organics, they also observed an unknown broad double band around 660 cm^−1^ that they could justify just by a structural disorder. In that paper, with the addition of different analytical techniques (that is, optical microscopy, scanning electron microscopy and energy dispersive X-ray spectrometry (SEM–EDS), Raman spectroscopy and time-of-flight secondary ion mass spectrometry (TOF–SIMS)), they found strong hints that those microorganisms participated actively in the synthesis of the microstromatolites. Raman laser damage can cause baseline perturbation by sample structural deformation, but the pictures taken with the microscope rule out this possibility (Fig. 3). Ruiz-Galende et al*.*^45^ studied samples from the Meñakoz outcrop, claiming the zone as a good Martian analogue. They measured hematite with the same band at 660 cm^−1^ that they also explained by asymmetries of the crystal structure caused by impurities in agreement with^44^.

Nevertheless, this broad feature at 660 cm^−1^ was unequivocally measured in all the samples with hematite associated to the 800 cm^−1^, regardless of the site and type of substrate. Thus, it can be characteristic of structurally altered hematite, which was also the predominant state of most of the collected samples.

#### Anatase

Like sulfur, anatase was also found in several samples at each site studied (see Supplementary Table S1). Anatase is a metastable mineral form of titanium dioxide (TiO_2_) very well characterized by Raman since, as mentioned before, its three distinctive peaks at 395, 510, and 635 cm^−1^ show strong intensity in this type of spectroscopy (Fig. 3). In the case of this mineral, we wanted to pay attention to the Raman intensity ratio between the three peaks because it can give information about structural modifications^46^. When comparing the Raman spectra of the anatase of N-IF-2, N-IF-3, and libraries minerals (Fig. 3), we could find different patterns in the Raman spectra depending on the origin of the mineral (generally volcanic, metamorphic, pegmatitic). In N-IF-2, the peak at higher frequencies (i.e., 635 cm^−1^) is the most intense. In N-IF-3, the peaks at 395 and 635 cm^−1^ have similar intensities, while the signal at 510 cm^−1^ almost vanishes.

In contrast, the anatase of the RRUFF database has the opposite Raman intensity trend than N-IF-2, that is, the peak at the lower frequency is the most intense. This mineral was found in Taftan (Pakistan), an andesitic stratovolcano^47^, which is part of a geothermal area with intense fumarolic activity and hot springs. As far as we know, there is no previous study analyzing this relationship between the Raman intensity signal of the three modes and the formation or mineral associations of the mineral. It is known that anatase is usually a secondary mineral derived from other titanium-bearing minerals. Also, it can be derived from the alteration of rocks by hydrothermalism or from crystal fractionation of igneous rocks, or be part of the precipitates of hydrothermal fluids from a metamorphic context at Earth. While the anatase from Taftan was potentially formed by magmatic processes, we hypothesize that in N-IF-2 and N-IF-3 it may be strongly affected by hydrothermalism, as the two samples were collected from currently inactive fumaroles. In N-IF-2, anatase was found together with hematite, whereas in N-IF-3 it appeared with quartz. The presence of quartz can be a sign of structural reordering of precipitated silica. Exist the possibility that the coexistence of anatase with diverse mineral associations influences its structure differently due to the introduction of distinct types of impurities. It is known that the level of impurities alters the structure and behavior of the mineral dramatically, for example, varying its transformation to rutile in a large temperature range from 550 to 1000$^\circ$C^48^. Impurities introduce crystal defects that are reflected in the Raman spectrum. From our data, we can speculate that differences in the Raman spectra can be related to impurities introduced by the other minerals accompanying the anatase during its formation or later alteration processes.

Thus, and considering that anatase is better detected by Raman spectroscopy than other mineral phases, and that it can even mask the others as shown in N-IF-3 with the quartz (Fig. 3b), with further studies analyzing the ratio between the three characteristic peaks it may be possible to infer the mineral assemblages present in a given sample. This observation is particularly relevant when we cannot perform XRD in our samples, as will be the case with the ExoMars rover.

A recent study speculates about the possible formation of anatase associated with microbial alteration in a volcano-glacial region in Iceland^49^. Nonoyama et al*.*^50^ conducted a conspicuous study compelling the observations pointed out in our research. They showed how it is possible to modify the mineral structure through biomineralization, exemplifying the process using anatase and a peptide as an organic template. They were able to control the structure and properties of anatase through this type of process. Glamoclija et al*.*^51^ found unusual anatase with rod morphology associated with fossil lipids. They related the mineral precipitation to low-temperature (below 100$^\circ$C) hydrothermal alteration with alkaline aqueous fluids. Very interestingly, in that work, they showed the Raman spectra of both “potentially biotic” rod-anatase and “abiotic” tetragonal-anatase (see Fig. 2 of^51^). The spectra of the two types of anatase displayed different patterns between the three characteristic peaks. The rod-shaped anatase exhibited the lowest signal at 510 cm^−1^, as in our sample N-IF-3, in agreement also with Bower et al*.*^52^, which grew anatase bio-induced by cyanobacteria at 70$^\circ$C (see Fig. 6c of^52^). However, the tetragonal-anatase displayed the most prominent signal at 635 cm^−1^ and a lower and similar intensity at 395 and 510 cm^−1^, as in our sample N-IF-2 (Fig. 3). Going back to Ruiz-Galende et al*.*^45^, in their samples from Meñakoz outcrop, they also found anatase which formation was suggested to be abiotic from the alteration of titanium‐rich minerals by hydrothermalism. They reported distribution patterns of the three anatase-characteristic peaks similar to those observed here for the N-IF-2 sample and associated by Glamoclija et al*.*^51^ with abiotic tetragonal anatase.

On the other hand, if confirmed, the presence of anatase on Mars could have very interesting implications due to its very-well photocatalytic characteristics. Also, its equilibrium with rutile can give hints about past formation conditions since, as mentioned, anatase transforms in rutile just at very high temperatures (above 550$^\circ$C^48^). Some interesting studies showed how the catalytic properties vary depending on the anatase–rutile proportions(^53^, and references therein). In this regard, experiments with anatase of application to Mars demonstrated that this mineral could be a catalyst to form chlorides and CH_4_ in the presence of CO_2_ and HCl under UV radiation^54^.

Our data revealed different patterns in the three peaks of anatase that suggest variability in the geological context where it was located. In this regard, we measured a characteristic pattern for each sample that, despite their similar geological substrate (both were taken from inactive fumaroles), showed different mineral assemblages (Fig. 3), which may imply differences in the rock formation and/or evolution over time. Thus, while hematite is characteristic of oxidized environments, quartz is formed from the crystalline realignment of silica formed after the acidic hydrothermal alteration of the primary lower Si-saturated basaltic rock. Silica is a phase relatively easy to detect during Mars in situ surveys, which finding may have important astrobiological implications. On the one hand, the hydrothermal spring scenario where silica can form imply the existence of favorable conditions for life, including nutrients and energy gradients. On the other hand, silica substrate is an excellent matrix for long-term biosignatures preservation^55,56^, so it is a prioritized target in missions searching for life.

#### Sulfates

Gypsum and Na-alunite were found in all the sites. Other types of sulfates, such as epsomite and halotrichite, were less abundant and just sampled in “Námafjall” (see Supplementary Table S1 online). In certain cases, Raman spectra of the natural samples are slightly distorted due to a poor degree in crystallinity and also because of the substitution of cations by others with different sizes inside the structure that promotes crystal defects observed as shifts of the Raman signals. In clay minerals, this process is recurrent. However, other mineral types also suffer this cation exchange^33^, such as the detected sulfate halotrichite (FeSO_4_·Al_2_(SO_4_)_3_·22H_2_O) that forms a solid solution with pickingerite (MgSO_4_·Al_2_(SO_4_)_3_·22H_2_O) where Mg^2+^ is substituted by Fe^2+^^57^.

**Figure 4:**
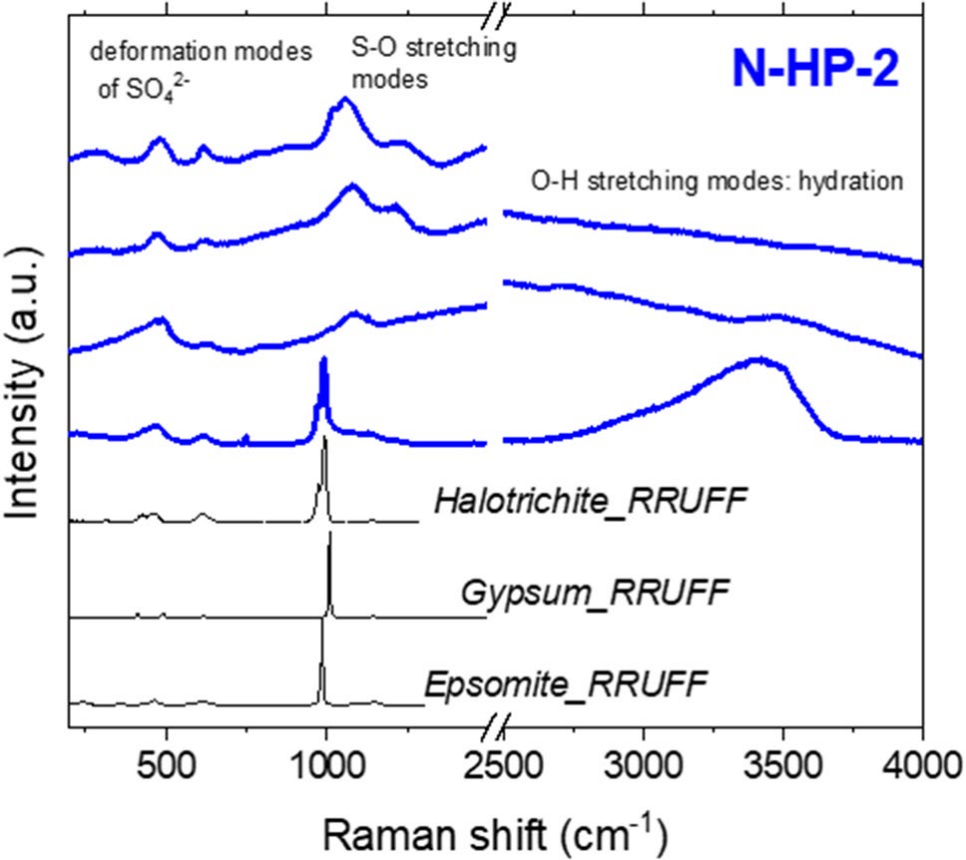
Raman spectra of the sample of the hot spring precipitate N-HP-2 composed of different types of sulfates. This spectroscopic technique allowed us to distinguish them (from the frequency shift around 1000 cm^−1^) and to study the hydration level (from the broad band at 3000–4000 cm^−1^ characteristic of the OH stretching modes of water).

In our samples, we generally observed a broadening of the band around 1000 cm^−1^, caused by the stretching of the SO_4_^2–^ molecule that indicates a partial loss of crystallinity (Fig. 4). As in the other mineral phases studied, we suggest that it would be possible to relate the amorphization not just to abiotic processes like the ones mentioned above but also to biomediation.

The detection of salts in a specific place indicates salty liquid water in that area, since they precipitate from aqueous solutions by evaporation or temperature decrease. An environment with water and elements that can act as nutrients, such as the ions that form salts, should always be considered as a potential astrobiological target. Given that the site where the samples in this work were collected from was rich in biomarkers^26^, and the measured Raman peaks were extremely distorted (i.e., wider and appearing slightly shifted to higher frequencies) with respect to the consulted database (assuming that they can be considered unaltered, or slightly altered, forms), the considered spectra of the Icelandic sulfates can be affected by biomediation, although abiotic sulfate can also precipitate in the studied regions^58,59^.

#### Clay minerals

**Figure 5:**
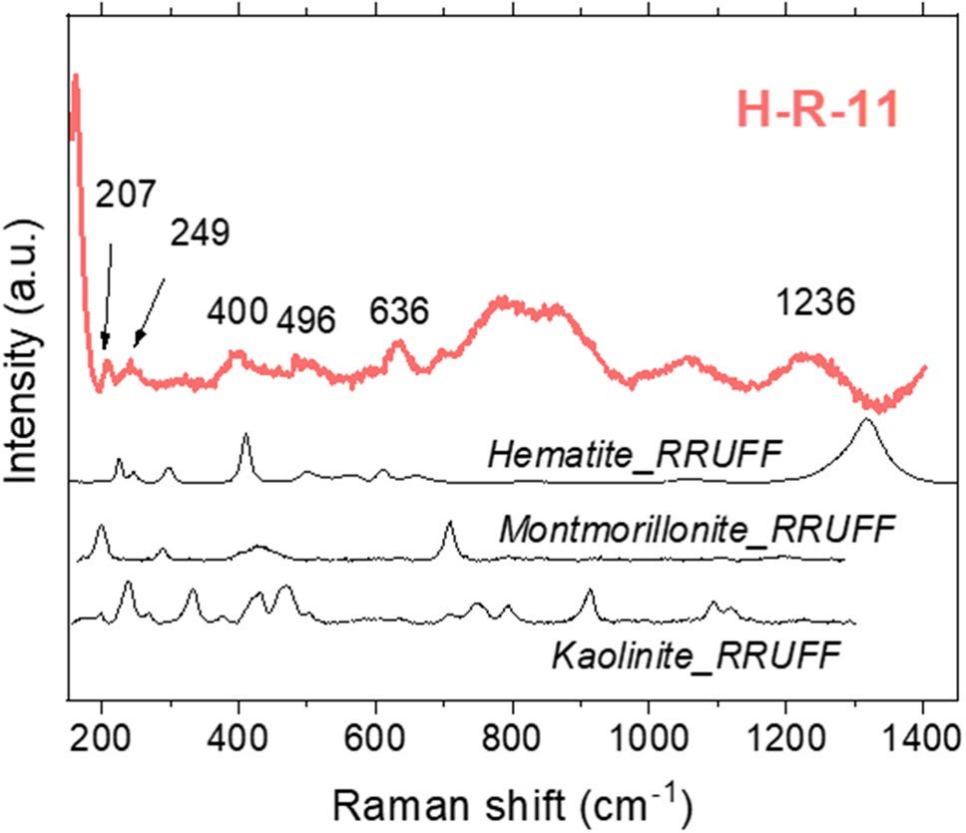
Raman spectrum of the rock sample H-R-11, where kaolinite, montmorillonite and hematite were potentially identified.

Clay minerals were detected by XRD in almost all the samples (see Supplementary Table S1 online). However, their measurement resulted very challenging with a non-confocal Raman spectrometer in the samples studied. Small grain sizes and high-level alteration decrease the Raman signal intensity and promote Raman shifts with respect to the databases, respectively. Therefore, their identification in the study case with the technical restrictions of the Raman spectrometer was not completely satisfactory. However, we could detect potential signals indicative of clay minerals in some samples, such as H-R-11 (Fig. 5). The XRD showed that the sample was composed mainly of kaolinite, montmorillonite and hematite. We could identify several mineral peaks by Raman spectroscopy, which correspond with the clay minerals, although some appear slightly displaced with respect to libraries. In this regard, Bathgate et al*.*^60^, who measured by Raman, IR and XRD Icelandic tephra samples, explained in their study the constraints and advantages of Raman spectroscopy against the other techniques. They concluded that some weak Raman signals not identified by any mineral of the reference RRUFF database could be caused by poor crystallinity of the minerals.

Clays are relevant for astrobiology since they can adsorb relatively small organic molecules in their interlayers under certain conditions and protect them from a hostile environment, such as the current surface of Mars. Furthermore, this adsorption of molecules is a reversible process. Thus, organics can be released if the environment changes and/or there are interactions with external agents such as microorganisms, although an unlikely large pH variation in the environment would be required to propitiate the change in the surface charges of the clays. Clay minerals are sensitive to both organic and inorganic molecules. It is difficult to determine the type of molecule adsorbed in the interlayers with Raman setups of laser spot size larger than the grain size of the minerals analyzed. However, from our previous works centered on the study of clay minerals, we know that its combination with IR spectroscopy can give satisfactory results^15,61^, providing the Raman technique powerful information on the surface state and the IR on the molecules adhered in the interlayer.

The Icelandic clay minerals were structurally altered, showing both spectroscopies the high level of amorphization of these phases.

Weiss et al*.*^62^ and Sheikholeslami & Lau^63^ relied on the notorious influence that bacteria metabolic activity has in the precipitation of minerals because it can alter the environment by oxidation/reduction of the molecules present. In the literature, we find disorder structures (in the form of Raman broad peaks) in fungi-like fossils associated with cavity-grown hydrothermal minerals^64^. It is possible even to relate the pattern of the Raman spectra with the alteration temperature at which the amorphization took place(^64^, and references therein). In this regard, Benison^65^ showed several Raman signatures that can indicate the presence of microorganisms, such as fluorescence, or the characteristic peaks of carotenoids (e.g., as the peaks found in some microbial mats in this work, see Fig. S2 of the Supplementary Information), or the peak around 2900 cm^−1^ corresponding to long-chain hydrocarbon waxes, or disorder graphite (Fig. 3 of^65^). Previously, Herbert et al*.*^66^ had observed how certain bacteria could cause the precipitation of poorly crystalline sulfides. From this observation, we speculate that microorganisms´ metabolic activities could disturb the growth of a crystal by utilization of lattice compounds, like S. In this line, a very recent study^67^ demonstrated that sulfur/sulfate-reducing bacteria could catalyze the precipitation of iron sulfide. We suggest that maybe the increase in the speed of some precipitation processes could have contributed to a decrease in the crystallinity of the biomediated minerals due to the lack of time for the new crystals to grow in the most orderly structure.

Although previous mineral libraries already include Raman spectra that provide information on some of the minerals of interest^27^, we wanted to perform a paired analysis with additional analytical techniques, and focus more deeply on Icelandic minerals which are scarcely included in the databases due to their usual high level of structural disorder.

The presence of microorganism cell components such as proteins (e.g., the amide I-characteristic C=O stretching around 1650 cm^−1^, or the amide III-characteristic band around 1240 cm^−1^), lipids (the characteristic CH_2_ bending vibrations at 1445 cm^−1^), or nucleic acids (e.g., the guanine and adenine nucleobases vibrations around the1570 cm^−1^), were not identified in any sample of the present study. CH stretching vibrations at 2925 cm^−1^, which are common of all the components, were not observed in any sample either^68^. Still, most of the samples showed high fluorescence due to the use of a green laser (of 532 nm of wavelength) that can be caused by microorganisms^65^. However, it was not possible to confirm the presence of biosignatures such as the organic molecules forming the cells components mentioned above with the analytical techniques used in this research. The lack of detection of biomolecule-related Raman signals could be due not only to fluorescence masking but also to a grain size smaller than the laser spot (i.e., 105 µm in our setup, see Methods section for the details about the Raman equipment and parameters used).

For mineral detection, XRD was successful in measuring crystalline phases. However, IR and Raman spectroscopies were also suitable for poorly-crystalline samples. As we have observed in this research, hydrothermal activity, can induce the formation of amorphous phases, and alter the primary mineral crystal structure due to high temperatures, the contact of chemically diverse fluids, and biomediation^26,64^. Thus, considering the occurrence of hydrothermal zones, Mars should be explored with techniques capable of characterizing both crystalline and amorphous phases as well, as is the case with Raman spectroscopy.

## Conclusions

The acidic and sulfur-rich environment found in Icelandic hydrothermal zones is considered a good analogue for local areas of past Mars. Silica, clay minerals, sulfur, sulfates, and oxides seemed to form during its history^3,10,11^ by different types of alteration processes. In a previous study, we explored molecular and isotopic biosignatures in different hydrothermal scenarios from Iceland with an analogy to Mars and described how the microbial activity is influenced by mineralogy and physicochemical parameters^26^. In this study, we aimed to move forward in understanding how microbial activity may imprint the minerals in a hydrothermal environment by studying the Raman spectra of 47 heterogeneous samples formed by different types of minerals (i.e., elemental sulfur, sulfides such as pyrite, sulfates such as gypsum and natroalunite, carbonates as calcite, oxides as anatase and hematite, both crystalline and amorphous silicates such as quartz and mineral clays, and opal and allophane, respectively).

The Raman spectra displayed peak shifts and broadening that reflected crystal structural modifications that could have been caused by weathering, hydrothermal and/or biomediation processes during their mineral history, which would be used as biosignatures. In particular, the detection of silica is especially interesting for astrobiology, as it cannot simply imply the past existence of a place rich in nutrients and redox gradients, but also this phase is known to be a good biosignatures protector^55,56^. Raman spectroscopy results in an excellent technique in the search for signatures indicative of biomediation, which may be accompanied by other analytical techniques for better interpretation. In this regard, ExoMars 2022 promises to provide successful results with the combination of three techniques, specifically, Raman and IR spectroscopies, and Gas Chromatograph-Mass Spectrometry.

## Methods

An Nd:YAG solid state laser with a wavelength of 532 nm non-polarized is used for Raman measurements, at power lower than 20 mW to avoid altering the minerals^31,69^. After focusing onto a monochromator (Horiba JobinYvon HRi550), with a diffraction grating of 1200 grooves/mm, the scattered light is detected with a charge coupled device (CCD) in “High gain” mode, with 1024 × 256 pixels cooled to 203 K for thermal-noise reduction. Fiber optics connect the spectrometer to a B&W Tek microscope with a 20 × objective (Microbeam S. A., Spain) with a spot size of 105 µm. Spectral resolution with a width slit of 200 µm results better than 5 cm^−1^. Spectral analysis was performed with OriginPro2015 software. The Raman spectra baseline was corrected using asymmetric least squares smoothing^70,71^. The bands were fitted to Lorentzian curves.

The infrared spectra were measured in the mid-infrared region (4000–400 cm^−1^) on a Thermo Nicolet Is50 FTIR spectrometer equipped with a Diffuse reflectance infrared Fourier Transform (DRIFT) using a DTGS-KBr detector and a XT-KBr beamsplitter at spectra resolution of 4 cm^−1^ and 64 scans.

X-Ray Powder diffraction data were collected on a Bruker D8 Eco Advance with Cu Kα radiation (λ = 1.542 Å) equipped with a Lynxeye XE-T linear detector. The X-Ray tube was set to 40 kV and 25 mA. The samples were scanned between 5° and 60° (2Ɵ) using a step size of 0.05° with a collection time of 1 s.

In Table S3 of the Supplementary Information the locations are indicated where each sample was collected from, together with a visual description of the field sites, as well as in situ temperature and pH measurements. Air temperature was measured with a Kestrel 5500 Weather Meter; soil temperature with a RS 1315 Wired Digital Thermometer with external thermocouple probe; and pH with a Hanna HI99121 Direct Soil Measurement pH Meter (photographs taken during the two field campaigns are shown in Table S4 of the Supplementary Information).

## Supplementary Information

Supplementary Information.
