# Supplementary material for: Raman spectroscopic peculiarities of Icelandic poorly crystalline minerals and their implications for Mars exploration
Source: Sci Rep. 2022 Apr 4;12:5640. doi: 10.1038/s41598-022-09684-x (PMC8979959; doi:10.1038/s41598-022-09684-x)
Supplement: Supplementary file 1 — Supplementary Information. [file 41598_2022_9684_MOESM1_ESM.pdf]

Raman spectroscopic peculiarities of Icelandic poorly crystalline minerals and their implications for Mars exploration

Victoria Muñoz-Iglesias*, Laura Sánchez-García, Daniel Carrizo, Antonio Molina, Maite Fernández-Sampedro, and Olga Prieto-Ballesteros

Centro de Astrobiología (CSIC-INTA), Madrid, Spain.

* Corresponding author: vmunoz@cab.inta-csic.es

Supplementary Information

Table S1. Mineralogy of the 47 samples collected, determined by XRD, IR and Raman spectroscopy.

Site	Type	Nomenclature	Elemental	Carbonates	Oxides		Silicates					Sulfates					Sulfides
			Sulfur	Calcite	Anatase	Hematite	Quartz	Opal	Allophane	Kaolinite	Montmorillo	Gypsum	Na-Alunit	Rozenite	Epsomite	Halotrichite	Pyrite
Hengill - Hveragerði																	
	rock	H-R-1		X						X	X						
	rock	H-R-2	X						X	X	X	X					
	rock	H-R-3	X					X		X		X					
	rock	H-R-4				X					X			X			
	rock	H-R-5	X	X						X	X						
	rock	H-R-6			X			X		X			X				
	rock	H-R-7			X			X		X			X				
	rock	H-R-8						X	X	X			X				
	mud	H-MP-1								X	X	X					X
	rock	H-R-9				X				X	X						
	rock	H-R-10				X				X	X						
	rock	H-R-11				X				X	X						
	rock	H-R-12			X		X			X							
	rock	H-R-13			X		X						X				
	rock	H-R-14					X				X						
	microbial mat	H-MAT-									X						
	microbial mat	H-MAT-				X											X
	microbial mat	H-MAT-	X														
	microbial mat	H-MAT-								X							
	microbial mat	H-MAT-								X							

	microbial mat	**H-MAT-**	**X**														
	microbial mat	**H-MAT-**	X														
	microbial mat	**H-MAT-**	X														
	microbial mat	**H-MAT-**						X			X	X					X
	microbial mat	**H-MAT-**	X							X							
**Krýsuvík - Seltún**																	
	rock	**K-R-1**				X				X							
	rock	**K-R-2**	X							X							
	rock	**K-R-3**			X					X							
	rock	**K-R-4**		X					X	X	X						
	rock	**K-R-5**								X			X				
	rock	**K-R-6**	X		X			X			X						
	rock	**K-R-7**	X		X					X	X						X
	rock	**K-R-8**				X				X	X						
	microbial mat	**K-MAT-**	X					X		X							X
	microbial mat	**K-MAT-**								X							
	hot spring	**K-HP-1**			X												
	active fumaro	**K-AF-1**			X					X			X				
	active fumaro	**K-AF-2**	X		X												
	mud pot	**K-MP-1**	X							X							X
**Námafjall**																	
	inactive	**N-IF-1**	X		X			X	X	X							
	inactive	**N-IF-2**			X	X		X		X			X				
	inactive	**N-IF-3**	X	X	X		X			X							
	inactive	**N-IF-4**	X														
	mud pot	**N-MP-1**	**X**		X												X
	mud pot	**N-MP-2**	**X**														
	hot spring	**N-HP-1**															
	hot spring	**N-HP-2**			X							X			X	X	

Table S2. Nomenclature equivalence of the samples analyzed both in this work and in our previous study focused on the fingerprinting molecular and isotopic biosignatures [26].

**Sample ID in [26]**	**Sample ID in this work**
**Hengill - Hveragerði**	
MAT-54	H-MAT-1
MAT-70	H-MAT-2
MAT-78	H-MAT-3
**Námafjall**	
IF-74	N-IF-1
IF-66	N-IF-4
IF-49	N-IF-2

IF-20	N-IF-3
MP-74	N-MP-1
**Krýsuvík - Seltún**	
AF-90	K-AF-1
AF-25	K-AF-2
MP-87	K-MP-1

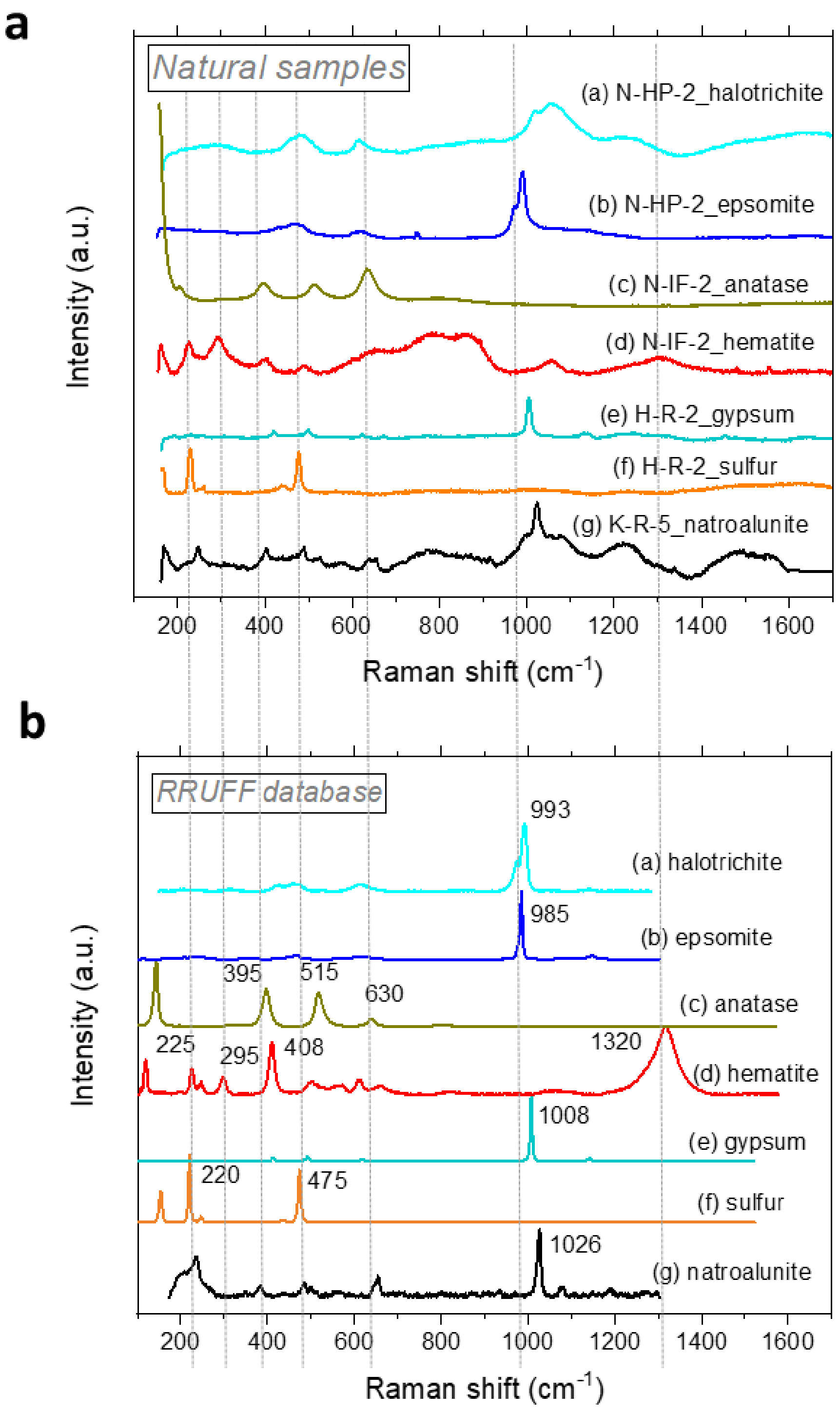

Fig. S1. Raman spectra of selected mineral phases identified in a) the Icelandic samples and b) minerals from the RRUFF database [27].

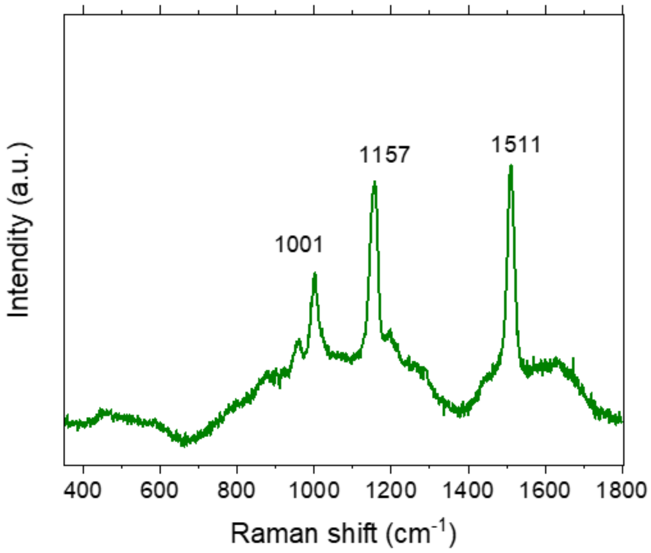

Fig. S2. Example of the chlorophyll Raman spectrum found in the green spots of the microbial mat H-MAT-1.

Table S3. Sample location coordinates, field sites description, and *in situ* temperature and pH measurements. "n.m." means not measured due to the sample nature or logistic limitations.

**Sample ID**	**Latitude, Longitude**	**Field site description**	**Air / soil temperature**	**pH**
**H-R-1**	64° 01.219 N, 21° 23.642 W	Dark and ochre-colored soil in hot ground	12.0° / 93.0°	n.m.
**H-R-2**	64° 01.219 N, 21° 23.642 W	Orangish efflorescence in hot ground	11.8° / 77.8°	2.83
**H-R-3**	64° 01.219 N, 21° 23.642 W	Grayish-white efflorescence in hot ground	11.8° / 93.6°	5.21
**H-R-4**	64° 01.236 N, 21° 23.597 W	Dark-colored rock	11.2° / n.m.	n.m.
**H-R-5**	64° 01.236 N, 21° 23.597 W	Dark-colored rock	11.2° / n.m.	n.m.
**H-R-6**	64° 01.217 N, 21° 23.645 W	Yellowish soil in hot ground	7.9° / 60.0°	1.50
**H-R-7**	64° 01.217 N, 21° 23.645 W	Salmon-colored soil in hot ground	7.9° / 46.0°	1.68
**H-R-8**	64° 01.219 N, 21° 23.642 W	Grayish-white efflorescence	11.8° / n.m.	n.m.
**H-MP-1**	64° 03.467 N, 21° 13.637 W	Hot pool mud below pale efflorescence	5.9° / 73.9°	2.52
**H-R-9**	64° 03.448 N, 21° 14.252 W	Brownish soil in inactive fumarole	4.7° / 5.8°	5.67
**H-R-10**	64° 03.448 N, 21° 14.252 W	Reddish soil in inactive fumarole	4.7° / 7.2°	4.25

**H-R-11**	64° 03.450 N, 21° 14.243 W	Brownish soil in inactive fumarole	4.7° / 5.8°	2.95
**H-R-12**	64° 03.436 N, 21° 14.232 W	Light-colored soil in active fumarole	4.6° / 83.5°	2.59
**H-R-13**	64° 03.436 N, 21° 14.232 W	Light-colored sinter	4.6° / n.m.	n.m.
**H-R-14**	64° 03.436 N, 21° 14.232 W	Light-colored sinter	4.6° / n.m.	n.m.
**H-MAT-1**	64° 01.205 N, 21 23.657 W	Dark green mat in hot pot side	11.8° / 54°	6.00
**H-MAT-2**	64° 01.204 N, 21 23.690 W	Pale green mat in river side	11.8° / 70°	6.00
**H-MAT-3**	64° 01.204 N, 21 23.690 W	White fibers in river side	1.8° / 78°	6.00
**H-MAT-4**	64° 01.221 N, 21 23.653 W	Greenish microbial mat in river shore	11.2° / n.m.	7.40
**H-MAT-5**	64° 01.221 N, 21 23.653 W	Ochre orangish microbial mat in river shore	11.2° / n.m.	6.33
**H-MAT-6**	64° 03.467 N, 21° 13.637 W	Water from hot pool	5.9° / n.m.	6.03
**H-MAT-7**	64° 03.467 N, 21° 13.637 W	Grey mud from hot pool	5.9° / n.m.	6.03
**H-MAT-8**	64° 03.436 N, 21° 14.232 W	Biofilm from hot spring	4.6° / n.m.	7.46
**H-MAT-9**	64° 03.436 N, 21° 14.232 W	Mud from hot pool	4.7° / 7.2°	4.25
**H-MAT-10**	64° 03.467 N, 21° 13.637 W	Microbial mat	5.9° / 73.9°	2.52
**K-R-1**	63° 53.848 N, 22° 02.920 W	Reddish soil in inactive fumarole	7.6° / 8.9°	5.27
**K-R-2**	63° 53.850 N, 22° 02.935 W	Greyish soil in active fumarole	7.6° / 21.6°	2.95
**K-R-3**	63° 53.851 N, 22° 02.935 W	Yellowish soil in active fumarole	8.8° / 19.9°	2.85
**K-R-4**	63° 53.573 N, 22° 03.906 W	Dark grey rock	3.5° / n.m.	n.m.
**K-R-5**	63° 53.690 N, 22° 03.314 W	Pale sediment near river	5.0° / 4.9°	3.65
**K-R-6**	63° 53.690 N, 22° 03.307 W	Yellowish soil near mud pot	5.0° / 30.0°	1.99
**K-R-7**	63° 53.745 N, 22° 03.262 W	Grey mud pot fluid	5.7° / 61.7°	3.70
**K-R-8**	63° 53.752 N, 22° 03.195 W	Dark grey soil near mud pot	5.7° / n.m.	n.m.
**K-MAT-1**	63° 53.859 N, 22° 02.938 W	Microbial mat	5.0° / n.m.	2.95
**K-MAT-2**	63° 53.859 N, 22° 02.938 W	Microbial mat	5.0° / n.m.	n.m.
**K-HP-1**	63° 53.859 N, 22° 02.938 W	from hot spring precipitation	5.0° / n.m.	2.73
**K-AF-1**	63° 53.708 N, 22° 03.302 W	White-pale yellow soil from fumarole hot mouth	11.7° / 90°	1.00
**K-AF-2**	63° 53.709 N, 22° 03.303 W	White-pale yellow soil from fumarole cold low part	11.7° / 25°	2.00
**K-MP-1**	63° 53.712 N, 22° 03.316 W	Pale grey mud pot fluid	11.7° / 87°	2.00

**N-IF-1**	65° 38.392 N, 16° 48.694 W	Pale yellow solid material from inactive fumarole	9.3° / 74°	1.00
**N-IF-2**	65° 38.392 N, 16° 48.695 W	Grey solid material from inactive fumarole	9.3° / 49°	4.00
**N-IF-3**	65° 38.392 N, 16° 48.696 W	Red solid material from inactive fumarole	9.3° / 20°	3.00
**N-IF-4**	65° 38.392 N, 16° 48.697 W	Ochre solid material from inactive fumarole	9.3° / 66°	3.00
**N-MP-1**	65° 53.359 N, 16° 48.594 W	Dark grey mud pot fluid	9.3° / 74°	2.00
**N-MP-2**	65° 53.359 N, 16° 48.595 W	Pale grey mud pot fluid	9.3° / 63°	n.m.
**N-HP-1**	65° 53.708 N, 22° 03.302 W	Hot spring precipitation	9.3° / n.m.	n.m.
**N-HP-2**	65° 53.708 N, 22° 03.303 W	Hot spring precipitation	9.3° / n.m.	n.m.

Table S4. Photographs of most of the studied samples (n=39) taken in the field during the two sampling campaigns. With consent for publication of identifying images.

**H-R-1**	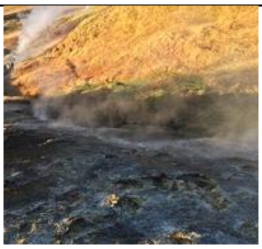	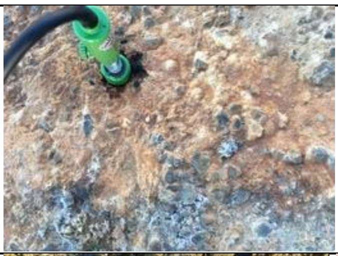
**H-R-2**	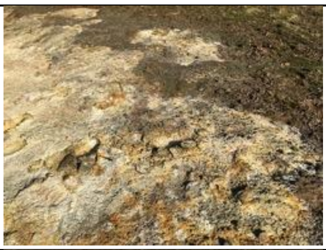	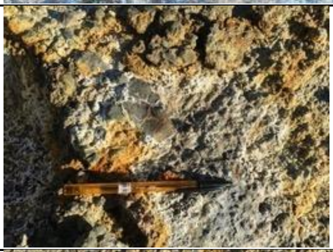 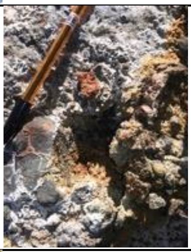
**H-R-3**	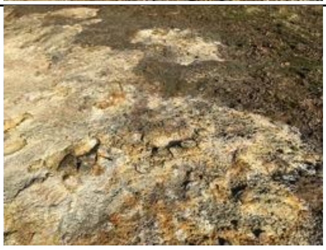	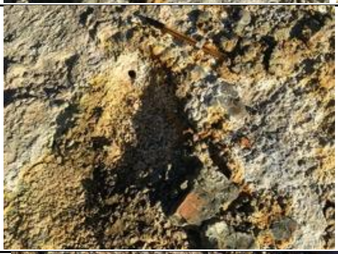
**H-R-4**<br>**H-R-5**	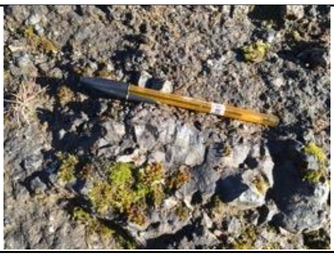	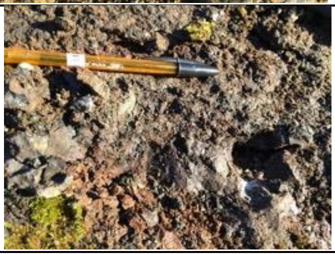

**H-R-6**	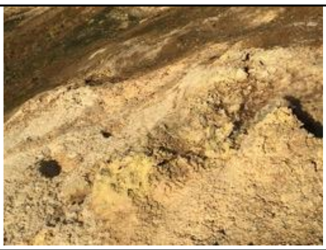	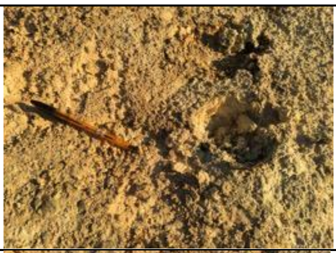
**H-R-7**	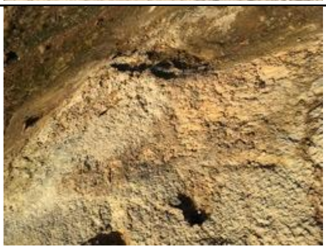	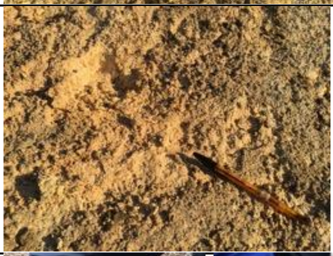
**H-R-8**	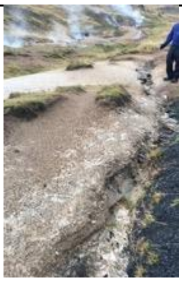	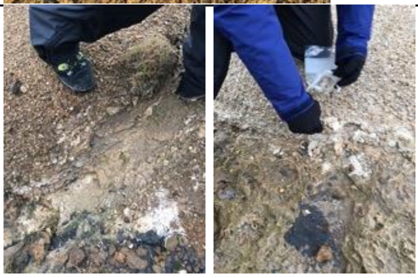
**H-MP-1**	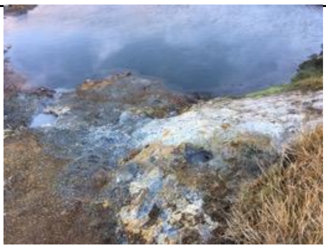	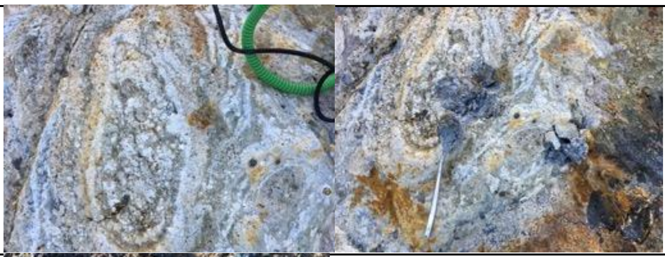
**H-R-9**	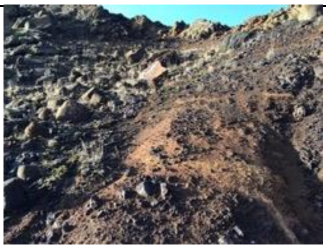	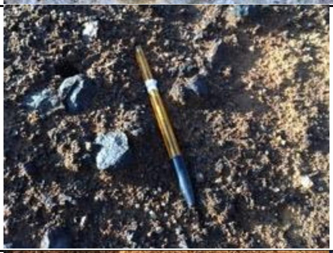
**H-R-10**	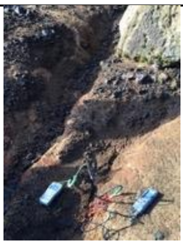	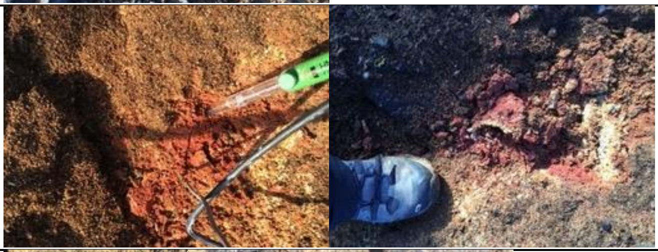
**H-R-11**	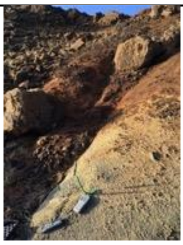	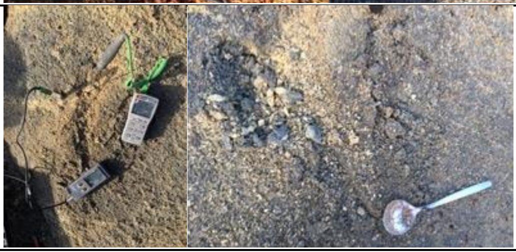

**H-R-12**	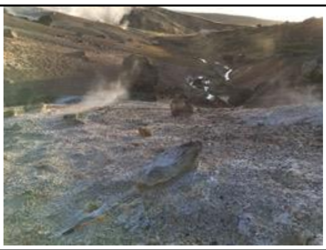	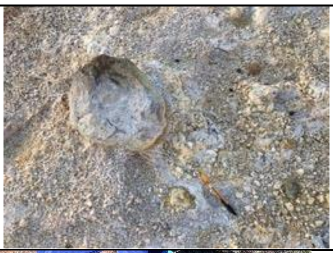
**H-MAT-1**	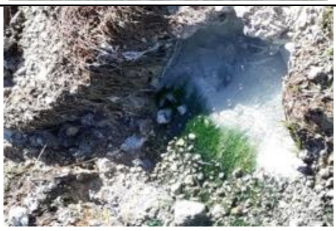	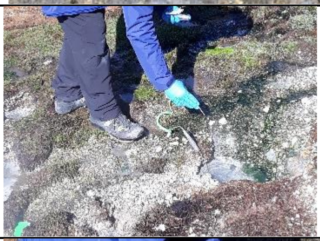
**H-MAT-2**	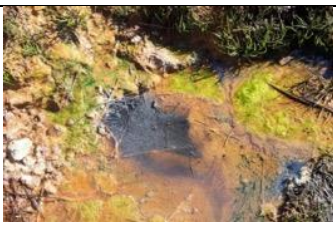	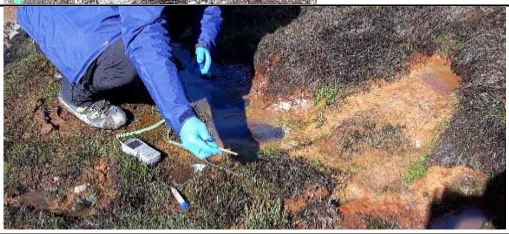
**H-MAT-3**	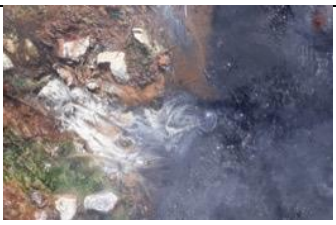	
**H-MAT-4**	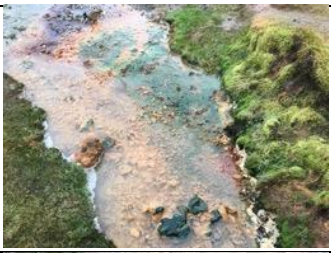	
**H-MAT-5**	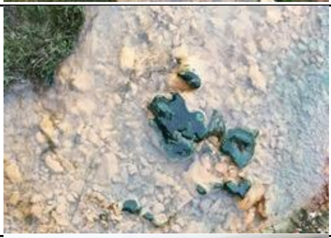	
**H-MAT-7**	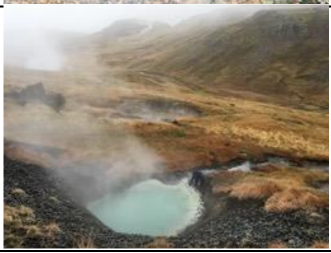	

**H-MAT-8**	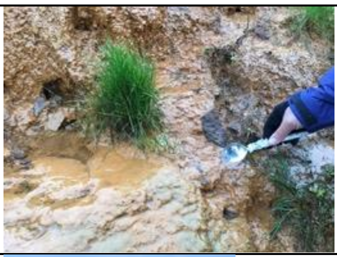	
**H-MAT-9**	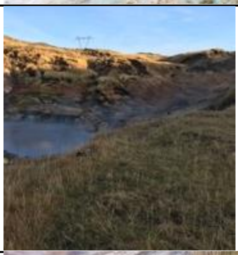	
**H-MAT-10**	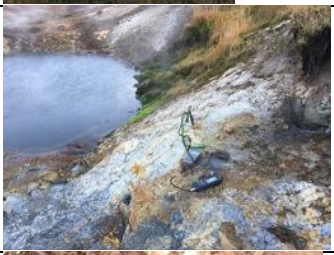	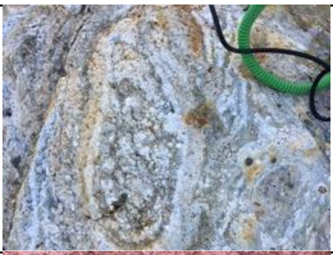
**K-R-1**	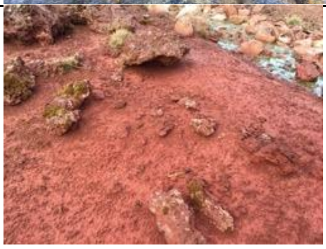	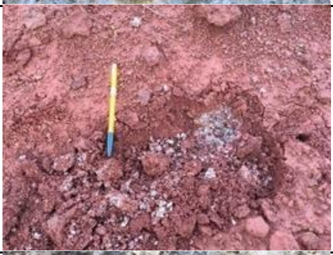
**K-R-2**	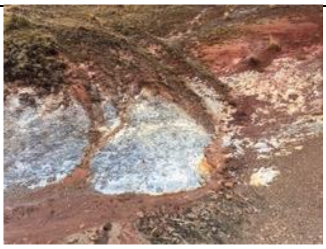	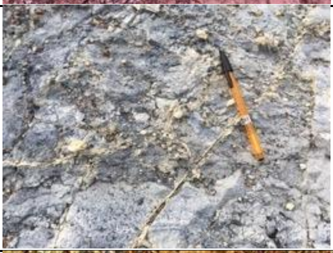
**K-R-3**	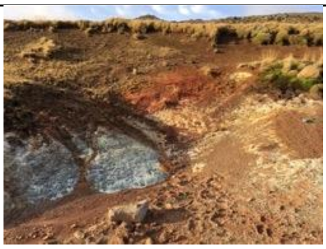	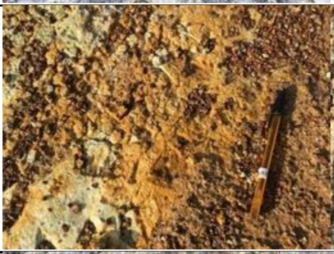 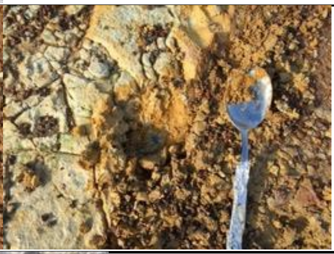
**K-R-4**	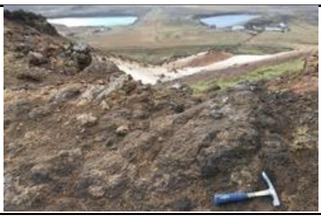	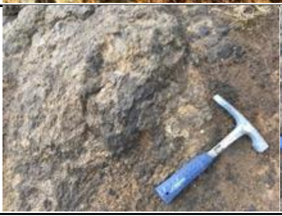 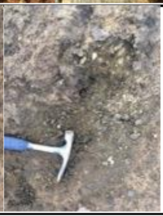

**K-R-5**	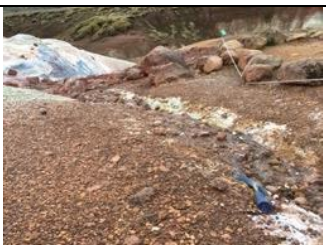	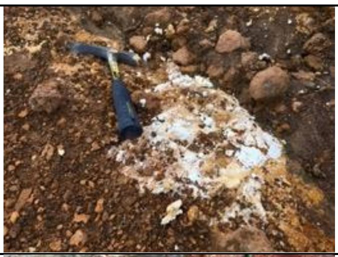
**K-R-6**	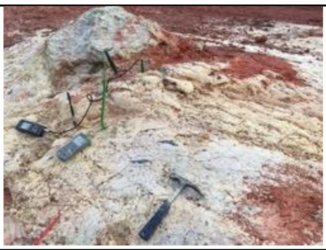	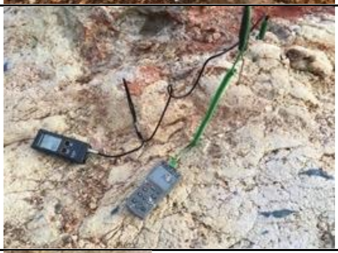
**K-R-7**	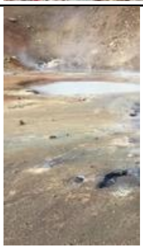	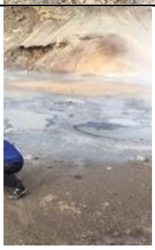
**K-R-8**	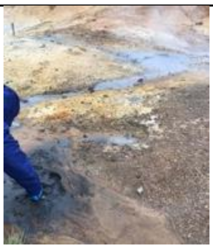	
**K-AF-1**	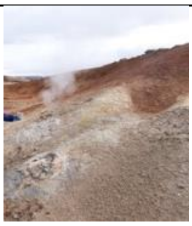	
**K-MP-1**	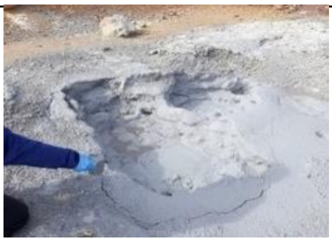	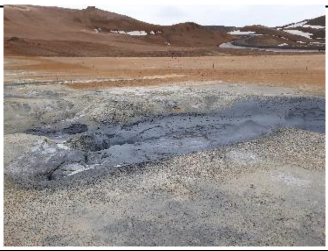
**N-IF-1**	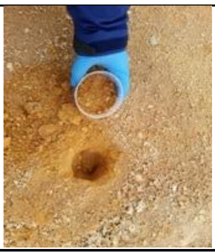	

**N-IF-2**	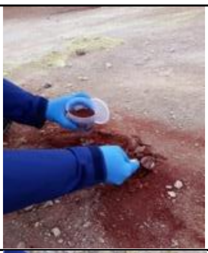	
**N-IF-3**	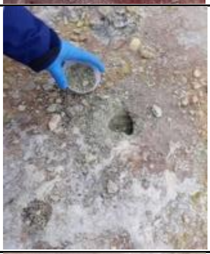	
**N-IF-4**	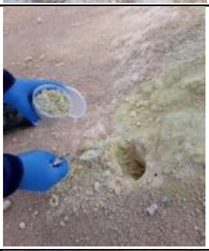	
**N-MP-1**	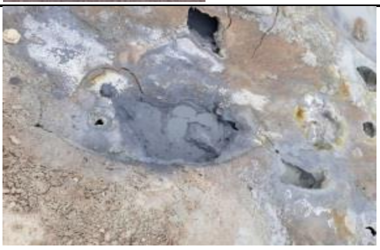	
**N-MP-2**	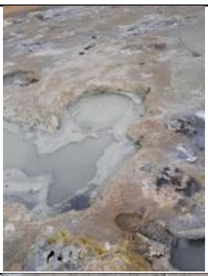	
**N-HP-1**	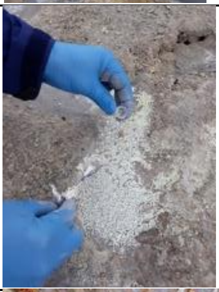	
**N-HP-2**	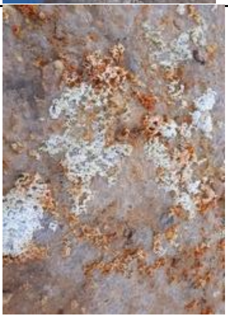	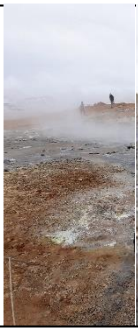 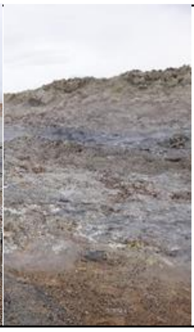
